# From legacy to innovation: A comprehensive review of vaccine platforms against viral infections

**DOI:** 10.1016/j.virusres.2026.199730

**Published:** 2026-04-16

**Authors:** Niloofar Farsiu, Fatemeh Khodadadpour Mahani, Nasir Arefinia, Javad Charostad, Mohammad Pardeshenas, Habibollah Mirzaei, Mohsen Nakhaie, Pouya Hassandarvish, Sazaly AbuBakar

**Affiliations:** a Gastroenterology and Hepatology Research Center, Institute of Basic and Clinical Physiology Sciences, Kerman University of Medical Sciences, Kerman, Iran; b Research Center of Tropical and Infectious Diseases, Kerman University of Medical Sciences, Kerman, Iran; c Bio Environmental Health Hazards Research Center, Jiroft University of Medical Sciences, Jiroft, Iran; d Department of Microbiology, Faculty of Medicine, Shahid Sadoughi University of Medical Sciences, Yazd, Iran; e Department of Microbiology, School of Medicine, Kerman University of Medical Sciences, Kerman, Iran; f Hepatitis Research Center, Deputy of Research, Lorestan University of Medical Sciences, Khorramabad, Iran; g Clinical Research Development Unit, Afzalipour Hospital, Kerman University of Medical Sciences, Kerman, Iran; h Tropical Infectious Diseases Research & Education Centre, Universiti Malaya, Kuala Lumpur, Malaysia

**Keywords:** mRNA vaccines, Viral vector vaccines, Virus-like particles, Vaccination/methods, Nanoparticles, Plants

## Abstract

Viral infections continue to pose a substantial global health concern, resulting in extensive morbidity and mortality among various populations. Although conventional vaccinations have been pivotal in managing and eliminating certain viral infections, the introduction of new viruses and the resurgence of existing ones underscore the ongoing necessity for creative immunization techniques. This review offers an extensive examination of both conventional and novel vaccine platforms for preventing viral diseases. It analyzes traditional approaches such as inactivated and attenuated vaccines in conjunction with innovative technologies, including subunit, viral vector-based, DNA, mRNA, and virus-like particle (VLP) vaccines. Furthermore, novel strategies to enhance vaccination efficacy, including nanoparticle-based and plant-derived vaccines, are examined. Each platform is evaluated according to its mode of action, immunogenicity, safety, scalability, and adaptation to novel threats. Empirical instances, especially observations from the COVID-19 pandemic, are employed to underscore the achievements, obstacles, and pragmatic utilization of these tools. By reviewing the scientific foundations and developmental pathways of diverse vaccine strategies, this paper offers a more profound understanding of the evolving landscape of viral vaccine development. Finally, this review emphasizes the critical role of innovation in strengthening global readiness for current and future outbreaks.

## Highlights

• Vaccine innovations enable rapid adaptation to emerging viral threats.
• Innovation also upgrades established platforms, not only creating new ones.
• Plant-made antigens/VLPs support low-cost production and improved stability.
• Each platform shows distinct advantages and challenges for safety and immunity.

## Graphical abstract

**Figure:**
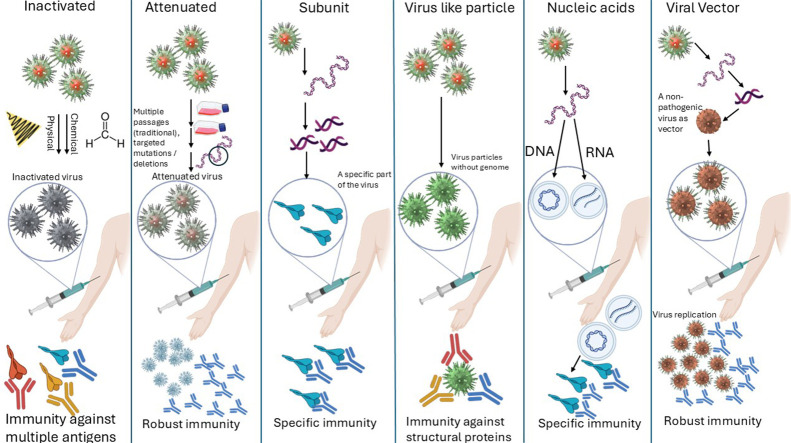

## 1 Introduction

Viruses represent the most widespread and numerous forms of infectious agents (Harper et al., 2020). As of 2019, over 1000 distinct viruses have been identified as capable of infecting humans (Lasso et al., 2019). These pathogens remain the predominant contributors to a wide range of human diseases. The development of clinical illness subsequent to a viral infection is influenced by factors inherent to both the virus and the host organism (Chaturvedi et al., 2004). Viral infections can affect various tissues and organs across the human body. These include the upper respiratory tract and lungs, which can be compromised by pathogens such as rhinoviruses, the influenza virus, and coronaviruses (Harper et al., 2020; Van Doorn and Yu, 2020). Additionally, viruses like Epstein-Barr virus (EBV) and rotavirus can target the gastrointestinal tract (Harper et al., 2020; Ignatova et al., 2020), while the liver is susceptible to infections from viruses such as the hepatitis B virus (HBV) (Harper et al., 2020). Poliovirus and coxsackievirus can impact the nervous system (Wouk et al., 2021). Furthermore, the Ebola virus may infect vascular endothelial cells, and white blood cells are vulnerable to infection by the human immunodeficiency virus (HIV) (Harper et al., 2020). Moreover, vector-borne viral infections, including dengue fever, yellow fever, West Nile fever, Zika, and chikungunya, have disseminated worldwide, exerting a profound influence on public health (Trovato et al., 2020). Throughout history, viral infections have accounted for a significant number of fatalities among humans. From smallpox, which was declared eradicated in 1980 and is estimated to have resulted in 300 to 500 million deaths during the 20th century (Thèves et al., 2014), to severe acute respiratory syndrome coronavirus 2 (SARS-CoV-2), which has infected over 600 million individuals and caused approximately seven million deaths globally (World Health Organization (WHO), 2023). These records and the alarming spread of aforementioned viral infections have prompted the scientific community to advocate for urgent measures aimed at the prevention and treatment of these emerging infectious diseases.

Vaccination is widely regarded as one of the most potent methods for stimulating the immune system to mount protective responses against infectious agents, thereby decreasing both morbidity and mortality rates (Trovato et al., 2020). The efficacy of vaccines in addressing various diseases is evidenced by notable successes, such as the complete eradication of smallpox, substantial advancements toward the elimination of polio, and significant reductions in measles-related fatalities, among other accomplishments (Kieny and Girard, 2005; Raisi Sarbijan et al., 2025). Recent advancements in technology have opened up possibilities for novel vaccine platforms that could offer solutions for viral pathogens lacking existing interventions.

At present, we are experiencing a turning point in the era of vaccination. On the one hand, the battle against viral infections is accelerating, and the need for rapid vaccine design, evaluation, and deployment has never been greater. This makes choosing the right vaccine platform for each pathogen more challenging because many viral infections have distinctive transmission dynamics, tissue tropism, and pathological patterns (Wang et al., 2024). Vaccine development must account for these differences to achieve effective protection while minimizing inefficient trial-and-error approaches. On the other hand, public confidence in vaccination is increasingly fragile, and vaccines are often judged under intense scrutiny for even rare adverse events, making it essential to prioritize platforms and design strategies that maximize safety, reproducibility, and transparency alongside effectiveness. In this context, innovation should not be viewed as “new platforms” alone (Brumbaugh et al., 2025). The most meaningful advances arise from integrating antigen engineering, delivery and formulation technologies, and manufacturing agility across platforms to optimize speed, durability, and safety. Finally, emerging tools such as 3D structural modeling, structure-guided antigen design, and other computational and in silico approaches can help predict immunogenicity and safety risks earlier, improving decision-making before in vivo studies and clinical trials and thereby supporting a more efficient and ethically grounded development pipeline (Bedi et al., 2023; Hsiung et al., 2024; Stiefel et al., 2023).

Across vaccine approaches, the quality of immunity is shaped by where and how the antigen is encountered by the immune system. Antigens delivered as proteins (or released extracellularly) are typically processed through the exogenous pathway and presented on MHC II, supporting CD4⁺ T-cell help and antibody maturation, whereas platforms that drive intracellular antigen expression (vectors, DNA, mRNA) more readily engage MHC I presentation and CD8⁺ T-cell priming. Cross-presentation can bridge these pathways but varies with formulation, adjuvants, and target cells. In addition, innate immune activation—provided by adjuvants, vectors, or nucleic-acid sensing—strongly influences response magnitude and polarization (Li et al., 2025; Pollard and Bijker, 2021). For this reason, platform selection is not only a manufacturing or safety decision, but also a mechanistic determinant of whether humoral, cellular, or mucosal immunity is preferentially emphasized.

In this review, we summarize the major viral vaccine platforms and highlight how recent innovations in antigen design, delivery and formulation, and manufacturing are reshaping their performance. We then compare platforms using shared criteria, including speed of development, safety, durability of immunity, scalability, and re-dosing feasibility, and conclude with cross-cutting challenges and future directions for next-generation viral vaccines.

## 2 Conventional vaccine platforms

### 2.1 Inactivated vaccines

Inactivated vaccines are produced from pathogens that have been rendered noninfectious. This inactivation is achieved through various chemical or physical techniques or a combination of both. Several chemical agents involved in this inactivation process include formaldehyde, glutaraldehyde, hydrogen peroxide, and beta-propiolactone (Ghattas et al., 2021). Among these agents, formaldehyde and β-Propiolactone have been extensively utilized for the inactivation of licensed human viral vaccines for years (Sanders et al., 2014). Physical inactivation is generally achieved through methods such as heating and/or pH denaturation. It also includes exposure to ultraviolet light and/or gamma irradiation (Ghattas et al., 2021). Well-known examples of vaccines developed using the inactivation platform encompass the poliovirus, hepatitis A virus, influenza virus, Japanese encephalitis virus, TBEV, and rabies virus (Ghattas et al., 2021; Sanders et al., 2014).

Inactivated vaccines are considered safe as the inactivation process inhibits replication and mutation. This prevents a return to virulence. These vaccines elicit a comprehensive immune response by utilizing the entire pathogen for immunization. As a result they target multiple antigens and mostly trigger humoral immunity. These vaccines typically do not require refrigeration, allowing for straightforward storage and transportation in a freeze-dried state. This characteristic contributes to their cost-effectiveness and enhances accessibility for populations in developing nations. However, a significant limitation of this vaccine technology is its reduced duration of immunity and capacity to stimulate cellular immune responses against intracellular pathogens. Subsequently, to achieve sustained protection, these vaccines necessitate larger doses and frequent booster shots due to their relatively lower immunogenicity (Ghattas et al., 2021; Yadav et al., 2014). This could serve as a constraint on the deployment of inactivated vaccines in regions where individuals lack consistent access to healthcare services and are unable to receive booster shots as scheduled (Yadav et al., 2014). Moreover, elevated dosages and frequent administration heighten the likelihood of adverse events, escalate manufacturing expenses, and diminish vaccine adherence. Methods of chemical and physical inactivation depend on experimental optimization of various parameters to attain a balance between effective inactivation and immunogenic response. This extended development timeline consequently leads to increased research and production costs, thereby hindering the ability to respond swiftly to emerging pathogens (Ghattas et al., 2021). Instances of inactivation being incomplete, the requirement of high biosafety levels for some highly pathogenic viruses, and the possible risk of causing antibody dependent enhancement (ADE) or original antigenic sin (OAS) due to the non-neutralizing or poorly neutralizing antibodies produced against non-conserved regions of the virus, have also been mentioned as drawbacks of inactivated vaccines which need to be addressed (Cutts et al., 2024; Herrera-Rodriguez et al., 2019; Kan and Li, 2023).

### 2.2 Attenuated vaccines

Attenuated vaccines are developed using pathogens that have been weakened to the extent that their virulence is significantly diminished, resulting in a loss of their major pathogenic characteristics. A crucial aspect of this technology is the ability to sustain the replication potential of the pathogens while preventing the onset of disease or a return to virulence (Ghattas et al., 2021). The attenuation process can happen through conventional or novel approaches. The traditional attenuation process involves the sequential transmission of a pathogenic agent through an atypical host. This prolonged passaging results in the acquisition of mutations within the new host. The resultant mutated pathogen exhibits substantial differences from the original pathogenic strain, rendering it incapable of causing disease in the original host while still effectively stimulating an immune response (Yadav et al., 2014). Recently, several innovative approaches for viral attenuation have emerged, particularly emphasizing genetic engineering techniques. One such method involves modifying the locations of synonymous codons to reconfigure the viral genome. This process of codon de-optimization has demonstrated a reduction in mRNA stability and translation efficiency, which subsequently leads to decreased protein synthesis, an increase in translational errors, and an overall attenuation of the modified virus (Groenke et al., 2020). A variety of licensed vaccines that have been created utilizing this platform include the measles, mumps, and rubella virus vaccine (MMR), the ZEBOV vaccine, the influenza vaccine, the rotavirus vaccine, and the smallpox (vaccinia) vaccine (Ghattas et al., 2021).

Attenuated vaccines can be relatively straightforward to develop for specific viruses. As these vaccines closely mimic a natural infection, they effectively stimulate the immune system, leading to robust cellular and humoral immune responses, frequently resulting in long-lasting immunity after just one or two doses. While the aforementioned advantages are notable, it is important to acknowledge the associated drawbacks. A significant concern regarding these vaccines is the potential for secondary mutations, which may result in a reversion to virulence, thereby posing a risk of disease. Consequently, immunocompromised individuals are advised against receiving these types of vaccines. Moreover, the empirical methods result in attenuation in ways that are random and unknown. For instance, the Japanese encephalitis virus (JEV) vaccine, which has been implemented since its approval in China in 1988, involves the SA14–14–2 attenuated variant which is non-neuroinvasive and non-neurovirulent. A recent separate study found the exact mutations in E, NS1/1′ and NS2A proteins are responsible for the loss of virulence. However, this mechanism was not known until now (Song et al., 2025). A further limitation associated with attenuated vaccines is their requirement for a cold chain to maintain efficacy, as well as the necessity for trained healthcare personnel to manage them. This situation incurs additional expenses that hinder mass immunization initiatives in developing nations, where access to reliable refrigeration is often inadequate. Moreover, the scarcity of qualified healthcare personnel further limits the extensive application of these vaccines (Yadav et al., 2014).

## 3 Subunit vaccines

### 3.1 Recombinant proteins and peptide/ multi-epitope constructs

The progress in biotechnology has enabled the development and production of defined viral antigens, including recombinant proteins and peptide-based constructs. Consequently, rather than employing the whole pathogen for immunization purposes, subunit components of antigens that are most effective in eliciting an immune response are utilized in vaccine development. This advancement has resulted in the emergence of a vaccine platform referred to as subunit vaccines. After identifying an immunoprotective antigen (and, in peptide or multi-epitope approaches, the relevant epitopes), subunit vaccines can be efficiently produced through either the production of the antigen followed by purification, or by utilizing recombinant DNA technology to generate antigen molecules from the viruses (Yadav et al., 2014). Currently, there are multiple subunit vaccines employed against viral infections, such as those designed for HBV, those targeting HPV, RZV (Shingrix) vaccine for varicella-zoster virus (VZV), the recombinant influenza vaccine (Flublok Trivalent), and respiratory syncytial virus (RSV) vaccines (Arexvy, Abrysvo) (Leung et al., 2024; World Health Organization (WHO), 2025, 2024; Yadav et al., 2014).

Subunit vaccines differ from inactivated or attenuated vaccines in that they primarily consist of specific antigenic fragments derived from viruses, without incorporating any infectious viral components. This characteristic alleviates concerns related to incomplete inactivation and the potential for virulence reversion. In contrast to inactivated and attenuated approaches, the lack of handling of the infectious virus makes the development procedure of subunit vaccines safer. Subunit vaccines are also regarded as safe, as they have a lower risk of potential harmful immune responses, which positions them as promising candidates for vaccination. Additionally, these vaccines can focus on specific, clearly defined neutralizing epitopes, potentially enhancing their immunogenicity and overall efficacy, and can induce strong humoral responses, while cellular immune responses are more reliant on the adjuvant usage (Wang et al., 2020). This vaccine platform, however, encounters several practical challenges. As previously noted, the identification of immunoprotective antigens and their corresponding antigenic epitopes is crucial for the advancement of these vaccines. This process of determining the most effective antigens that stimulate the immune response is both empirical and labor-intensive. Also, following the identification process, it is essential to purify significant antigens. Enhanced purification of the subunit components may result in a reduction of the immunogenicity of subunit vaccines, thereby restricting their applicability. This reduction can be counteracted by formulating subunit antigens with an adjuvant (Yadav et al., 2014).

### 3.2 Virus-like particle (VLP) vaccines

An additional platform of subunit vaccines that has garnered increased attention in recent years is virus-like particle (VLP) vaccines. These vaccines are constructed from proteins derived from viruses, which are organized to create a particle resembling the virus itself (Donaldson et al., 2018). VLP vaccines demonstrate many characteristics typical of conventional vaccines; however, they do not possess the capacity for replication due to the absence of a viral genome. This feature makes them a secure platform for the advancement of vaccine development (Mohsen et al., 2017). These vaccines are generally produced in bioreactors after the transfection of various cell types, including yeast, plant, insect, bacteria, or mammalian cells, with one or more genetic constructs. These constructs are designed to encode a minimum of two structural elements of the original virus, facilitating the self-assembly of particles (Ghattas et al., 2021).

Currently, several effective VLP vaccines have been developed for the prevention of HBV (Recombivax HB and Engerix-B) and HPV (Gardasil and Cervarix). Furthermore, numerous VLP vaccines are presently undergoing clinical trials aimed at combating various viral pathogens, including influenza A virus, chikungunya virus, and Zika virus (ZIKV) (Donaldson et al., 2018; Ghattas et al., 2021). VLPs mimic the structural characteristics of viruses, thereby eliciting both humoral and cellular immune responses. Additionally, they lack any viral genetic material, enhancing safety for both vaccine recipients and healthcare personnel involved in vaccine administration. This feature presents a significant benefit over traditional vaccines, including attenuated and inactivated forms, as it eliminates the risk of inadvertent infection (Fuenmayor et al., 2017). Also, the enhanced efficacy of this vaccine platform, in contrast to conventional vaccines, has been ascribed to its multivalent interactions and heightened avidity with innate immune system cells, leading to their subsequent activation. However, the practical application of this technology is hindered by several disadvantages, including manufacturing challenges related to vaccine design, purification, and storage, which also contribute to increased costs (Ghattas et al., 2021).

## 4 Nucleic acid-based vaccines

Since the inception of messenger RNA (mRNA) vaccines for infectious agents, notably the influenza virus in 1993, through the development of self-amplifying RNA (saRNA) vaccines utilizing alphaviruses, to the first global authorization of an mRNA vaccine for COVID-19 in 2020, viruses have consistently been intertwined with and a significant focus of this innovative technology (Pascolo, 2023; Zhou et al., 2023). The objective of mRNA vaccines is to provide RNA which leads to the synthesis of protein antigens, hence eliciting an immunological response and promoting the activation of innate, humoral, and cellular immunity against the antigen inside the target cell (Hajiaghapour Asr et al., 2023; Le et al., 2022). The advancement of lipid nanoparticles and their use in this technology offers protection of mRNA against enzymatic breakdown and improves vaccination effectiveness by enhancing cellular absorption. For instance, DLin-MC3 containing lipid nanoparticle exhibited robust immune responses to SARS-CoV-2 in vivo and many other approved vaccines against other viral agents also use nanoparticles as a delivery system (Wu et al., 2024; Yavuz et al., 2023).

Numerous mRNA vaccines have been developed targeting influenza viruses, human HIV-1, Nipah virus, rabies virus, and other viruses (Ladak et al., 2022; Rzymski et al., 2023). For example, Moderna RSV vaccine candidate, mRNA1345, encodes the RSV prefusion F glycoprotein, generating robust neutralizing antibody responses and demonstrating favorable tolerability in phase 1 studies (Zhou et al., 2023). Moreover, preclinical studies indicate that ZIKV mRNA vaccines, such as Moderna mRNA-1893, exhibit sustained immunity, while dengue virus (DENV) mRNA vaccines are in development to mitigate the hazards associated with antibody-dependent enhancement (ADE) (Chen et al., 2022). mRNA technology offers significant promise as a platform for preventing viral illnesses, and its independence from hazardous chemicals or cell cultures susceptible to adventitious viruses makes it a formidable competitor to conventional vaccinations. Unlike DNA vaccines, which have a theoretical risk of genomic integration because they must reach the nucleus, mRNA vaccines do not pose an integration risk. The high potency, safety, and efficacy, along with the capacity for swift clinical development and potential for rapid, cost-effective manufacturing, render these vaccines optimal platforms for designing preventive measures against viral infections, especially emerging and re-emerging viruses that necessitate prompt design and mass production (Gote et al., 2023; Hajiaghapour Asr et al., 2023; Ye et al., 2023). The effectiveness of this vaccine technique was shown when mRNA vaccines were created in an extraordinary timeframe of under one year and authorized for the COVID-19 pandemic (Tian et al., 2022; Zhou et al., 2023).

In addition to nonreplicating mRNA, saRNA and circular RNA (circRNA) represent significant RNA-based innovations for vaccine immunization presented to date (Le et al., 2022; Zhou et al., 2023). circRNA has a distinctive covalent circular structure that provides enhanced pharmacological and biological stability, safeguarding it against exonuclease breakdown. circRNA has been used as a vaccination strategy to address SARS-CoV-2. Qu et al., engineered a circRNA that encodes the trimeric receptor binding domain (RBD) for both Delta and Omicron variants (Qu et al., 2022). saRNA vaccines signify a more advanced development of the mRNA vaccination paradigm, designed to enhance RNA amplification inside host cells. This is accomplished by replicating components from alphaviruses, resulting in enhanced and sustained immune responses. McKay et al. created a saRNA vaccine that encodes a stabilized SARS-CoV-2 spike protein, demonstrating notable immunogenicity (McKay et al., 2020). saRNA-based vaccines have been examined in preclinical research for their efficacy in protecting against many viruses, including ZIKV, influenza, herpes simplex virus, HIV, and Ebola (Chen et al., 2022; Matarazzo and Bettencourt, 2023; Ye et al., 2023; Zhou et al., 2023). Despite progress, mRNA vaccines have obstacles such as the dangers of immunological responses, possible edema or thrombosis, instability and need for delivery systems, the need for cold chain transmission, and undetermined long-term adverse effects. The durability of immunological responses to these types of viruses also needs more study as they are novel approaches that have not been fully investigated. Nevertheless, high efficacy, swift developmental potential, and economical production position RNA-based vaccine technology as a crucial asset in addressing new and re-emerging viruses (Buhre et al., 2022; Gote et al., 2023; Hajiaghapour Asr et al., 2023; Liu, 2022).

DNA vaccines, a nucleic acid-based vaccine technology employed against viral infections, were first introduced in 1960. Their mechanism involves delivering a DNA plasmid containing the genes for desired viral antigens to the cell nucleus, thereby utilizing cellular functions for antigen synthesis, presentation, and recognition by the host immune system (Ali et al., 2023; Pagliari et al., 2023). For instance, a DNA vaccine that includes the M genome segment of the Andes virus (ANDV), a rodent-borne hantavirus, was created. This DNA vaccine encodes the viral envelope glycoproteins and effectively elicits potent immune responses against the virus (Paulsen et al., 2024). DNA vaccines have advantageous characteristics, including ease of storage, rapid and cost-effective mass manufacturing, and enhanced stability and safety compared to conventional methods for combating viral diseases (Paulsen et al., 2024; Rezaei et al., 2019; Ullas et al., 2014).

The DNA vaccine (pcDEST40-gpB) for infectious laryngotracheitis virus (ILTV) demonstrates superior protection and enhanced control of viral shedding compared to the attenuated TC-propagated ILT vaccine (Gamal and Soliman, 2023). The PETIM-pIRES-Rgp combination has shown equal and robust effectiveness in managing rabies compared to the inactivated viral vaccine (Rabipur) (Ullas et al., 2014). Additionally, DNA vaccines can include more genes, allowing them to elicit protection against several targets simultaneously. The pVAX-AG4-ub DNA vaccine, for example, contains the tick-borne encephalitis virus (TBEV) NS1, NS3, NS5, and E proteins (Kisakov et al., 2023). Although DNA vaccines provide an innovative method for preventing viral infections, some concerns associated with this technique have been noted. The primary concern is the safety of these vaccinations due to a small risk of genetic integration. Furthermore, anti-DNA immune responses have been shown in both clinical and preclinical investigations (Rezaei et al., 2019). The lack of optimal delivery techniques undermines the effectiveness of immunization (Lu et al., 2024). However, using nanoparticles and electroporation for DNA transport to cells has proven highly effective. For instance, complete protection and strong immunity against homologous IAV-S strain challenge in pigs have been shown after the intramuscular injection of a single dose of LNP-DNA (Nguyen et al., 2023; Rezaei et al., 2019). Additionally, it has been observed that cationic guanidium polymer-pDNA polyplex vaccinations are effective against Newcastle disease virus (NDV) infection in vivo (Luther et al., 2023).

The route of vaccine delivery plays a vital role in the efficacy of these vaccines. Kamili et al. demonstrated that, when delivered by gen gun, an anti-hepatitis E virus (HEV) DNA vaccine could protect Cynomolgus macaques against challenges with a heterologous HEV (a Mexican strain), while the intradermal immunization approach was ineffective (Kamili et al., 2004). Furthermore, certain DNA vaccines, despite their ability to stimulate an immune response, do not provide comprehensive protection against viral infection (Kisakov et al., 2023). An in vivo study showed that the DNA vaccination for canine distemper virus (CDV) enhanced pathogenicity but did not entirely inhibit viral reproduction, viremia, or virus shedding (Zhao et al., 2023). This has similarly applied to the AG0302-COVID-19 vaccine targeting SARS-CoV-2 (Nakagami et al., 2023). Vaccination using DNA vaccines against chronic viral illnesses such as HIV, HBV, and hepatitis C virus (HCV) has also been shown to be mostly ineffective (Liu, 2022). Despite ongoing controversies regarding the safety of DNA vaccines in human populations, these vaccines present significant opportunities to combat fatal viruses, potentially saving numerous lives. Crimean-Congo hemorrhagic fever virus (CCHFV) is a pathogenic tick-borne virus for which no official vaccines are available for public consumption. Nevertheless, preclinical research on DNA vaccines that encode nucleoprotein and glycoprotein precursor antigens of CCHFV has revealed promising results in the induction of both humoral and cellular immunity (Hawman et al., 2023).

## 5 Viral vector-based vaccines

Viral vector-based vaccines, first introduced in 1972, have emerged as a prevalent vaccine type for various infections, especially viral ones (Vanaparthy et al., 2021). Viral vector vaccines offer distinct advantages compared to alternative vaccine types. Nucleic acid-based vaccines are prone to degradation and necessitate an effective delivery system. Viral vector vaccines are not subject to this problem. Furthermore, in contrast to traditional vaccination approaches, these vaccines can elicit immune responses and inflammation independently of adjuvants (Trivedi et al., 2023). Viruses characterized by large packaging capacity, broad host range, the capacity to induce CD8+ T cell responses, and the absence of integration into the host genome are optimal candidates for developing viral vector vaccines. Numerous viruses, including adenoviruses (AdVs), adeno-associated viruses (AAVs), and HSV, have been utilized as vectors (Zeng et al., 2023).

Viral vector vaccines have been widely utilized, especially throughout the COVID-19 pandemic. The Johnson & Johnson Human Ad26 vaccine, the Oxford/AstraZeneca ChAdOx1 vaccine, and the Russian Sputnik V Ad26 and Ad5 vaccines employed non-replicating viral vector technology and exhibited high efficacy in human trials (Mudrick et al., 2022). Research indicates that a single administration of viral vector vaccines can provide substantial protection against various pathogenic viruses, including Ebola. An AdV-based vaccine obtained emergency approval during the 2014 Ebola outbreak. The rVSVΔG- ZEBOV vaccine demonstrated favorable outcomes following a single intramuscular administration in preclinical trials. The rVSV-ZEBOV vaccine induced anti-Ebola antibody responses in clinical trials despite causing transient rVSV viremia. Participants receiving a second dose demonstrated significantly enhanced immunity (Regules et al., 2017; Wang et al., 2023). A single dosage of VSV-based vaccines against various hemorrhagic fever viruses, including Marburg virus (MARV), Lassa virus (LASV), and CCHFV, has also shown high efficacy (Wang et al., 2023). Numerous viral vector vaccines utilize non-replicating viruses, which are incapable of replication and serve exclusively as delivery systems. Nevertheless, replicating viral vector vaccines have also been developed, which generate new viral particles through the infection of host cells (Kamel et al., 2023; Sasso et al., 2020; Trivedi et al., 2023; Vanaparthy et al., 2021; Zeng et al., 2023). A comparative study of replication-defective AdV and replicating single-cycle AdV (SC-Ad) vaccines expressing the SARS-CoV-2 spike protein indicated that the SC-Ad vaccine elicited a more robust and durable immune response (Mudrick et al., 2022). Arun V. Iyer et al. demonstrated that a replicative, non-neurotropic, highly attenuated gK-deleted HSV vaccine conferred lasting protection in mice against HSV-1 and HSV-2 genital infections following intramuscular administration (Iyer et al., 2013). A study demonstrated that a single injection of a replicating Venezuelan equine encephalitis virus (VEE)-based viral vector vaccine conferred protective immunity against DENV challenges in BALB/c mice (Khalil et al., 2014). The administration route and dosage of vaccination play a crucial role in determining the effectiveness of viral vector vaccines in managing viral infections. A study on recombinant AdV as a vaccine vector examined four doses and two vaccination routes against HCV. The findings indicated that the optimal intramuscular vaccine dose could induce significant inflammatory responses, along with strong humoral and cellular immunity in mice (Kamel et al., 2023; Singh et al., 2014). Viral vector vaccines also face important limitations that can affect both efficacy and safety perceptions. A key challenge is pre-existing immunity to vector backbones, which can blunt transduction and reduce vaccine immunogenicity. McDonald et al. (2021) reported that pre-existing neutralizing antibodies against viral vectors can substantially diminish immune responses against SARS-CoV-2 (McDonald et al., 2021). In addition, immunogenicity can be dose-dependent, creating a practical trade-off between achieving stronger responses and constraints related to tolerability and scalable manufacturing. Ledgerwood et al. (2017) showed that higher particle doses of Chimpanzee AdV vector Ebola vaccine generated stronger glycoprotein-specific antibody and T-cell responses than lower doses, also suggesting that the solution to pre-existing immunity against viral vectors can be the use of non-human viruses as vectors (Ledgerwood et al., 2017). Safety concerns have also influenced confidence in some vector-based approaches, including rare but serious events such as vaccine-induced immune thrombotic thrombocytopenia (VITT) reported in the context of certain adenoviral vector COVID-19 vaccines, underscoring the importance of careful risk–benefit assessment (Cines and Bussel, 2021). Finally, safety outcomes can be shaped by the delivery route and procedure rather than the vector itself. For example, Sobh et al. (2023) reported an 8% cumulative incidence of serious adverse events after ocular AAV gene therapy, noting that many were procedure-related rather than clearly attributable to the vector backbone (Sobh et al., 2023). Future research in viral vector vaccine design should prioritize developing tropism-specific vectors and optimizing these systems to elicit strong immune responses without any side effects. By customizing vectors to specifically target tissues or cells and optimizing their design, researchers can enhance immune protection against viral infections.

## 6 Innovations reshaping viral vaccine platforms

Although many vaccine candidates still fall within long-established platform categories, the transition from the traditional era to modern vaccinology has been driven by major innovations in how vaccines are designed, delivered, and manufactured. For this reason, and building on concepts introduced in earlier sections, we highlight key innovative strategies in this section. In particular, recent advances have transformed antigen design and optimization, delivery and formulation technologies, and manufacturing and scale-up. Together, these innovations enable faster and more cost-effective development, improve delivery and immunogenic performance, strengthen safety profiles and support more efficient and ethically grounded, preclinical and clinical evaluation.

### 6.1 Nanoparticle-based vaccination approaches

Numerous vaccine platforms necessitate effective delivery systems to guarantee targeted administration and protection of genetic materials from degradation. Nanoparticles are appropriate for this application because of their nanoscale sizes and distinct physicochemical characteristics (Hajiaghapour Asr et al., 2023; Tursi et al., 2023). They have been widely utilized in vaccines, with the BNT162b nanoparticle-based mRNA vaccine marking a breakthrough as the first approved example, triggering more focused research in this area (Wu et al., 2023). The interaction between nanoparticles and cellular mechanisms remains an area of ongoing research; however, these particles exhibit significant biocompatibility. In addition to their role as delivery vehicles, nanoparticles are utilized in both in vitro and in vivo development of nano-vaccines to induce immune responses. The in vivo approach is more cost-effective and facilitates rapid production in response to emerging viral epidemics (Farsiu et al., 2024; Hajiaghapour Asr et al., 2023; Tursi et al., 2023). A significant advantage of nanoparticles is their ability to carry multiple antigens, effectively addressing viral genetic and antigenic variability (Zhang and Cui, 2023). A multivalent epitope-based nanoparticle vaccine that includes highly conserved epitopes from influenza virus proteins—specifically the α-helix of hemagglutinin, the ectodomain of matrix protein 2, and HCA-2 of neuraminidase—exhibited universal protection against various influenza A and B viruses, showing promising results in mice for up to six months following vaccination (Pan et al., 2023). The SCTV01B nanoparticle vaccine, which integrates *Streptococcus pneumoniae* serotype 14 polysaccharides with the SARS-CoV-2 spike protein RBD, has also demonstrated extensive neutralization of all of the significant SARS-CoV-2 variants, including Omicron, as well as strong opsonophagocytic activity against S. pneumoniae in preclinical studies. The advancements highlight the potential of nanotechnology in developing universal vaccines for emerging infections (Deng et al., 2022).

### 6.2 Plant-based vaccination approaches

Recently, novel approaches to developing virus-like particle (VLP) vaccines have been made to address the downsides of this traditional technique. Plants are an alternative expression system for producing recombinant protein or VLP vaccines and can also be used as carriers and vaccine scaffolds. Plant virus-based vaccines can be generated via stable expression in transgenic or transplastomic plants or through techniques such as agroinfection or viral infection for transient expression. Various plant viruses, including the tobacco mosaic virus (TMV), cowpea mosaic virus (CPMV), potato virus X (PVX), alfalfa mosaic virus (AlMV), and papaya mosaic virus (PapMV), are being utilized in the development of novel vaccines (Ortega-Berlanga and Pniewski, 2022; Su et al., 2023). Preclinical studies indicate that malva mosaic virus (MaMV) presenting the canine influenza virus H3N8 antigen can confer protection against heterologous mouse-adapted influenza virus strains (Kheirvari et al., 2023). Plant-based vaccines offer an inexpensive approach, enabling the mass production of vaccines in plant-based facilities, such as greenhouses or open fields. Research indicates they can reduce certain biosafety and manufacturing concerns because they do not require propagation of human pathogens in production systems (Nikitin et al., 2023; Su et al., 2023). Moreover, these vaccines demonstrate prolonged storage capabilities, enhanced thermostability, and the potential for oral administration (Samanta and Chatterjee, 2022; Sohn et al., 2023). The vaccines primarily generate viral structural proteins that can elicit strong immune responses. Recombinant avian influenza virus and NDV proteins expressed in plants have been developed, demonstrating the capacity to form influenza HA-based VLPs and elicit robust humoral and cellular immunity (Nurzijah et al., 2022; Zahmanova et al., 2022). Furthermore, these vaccines demonstrate substantial protection against major flaviviruses, with no evidence of ADE following vaccination. Using targeted antigens, such as the West Nile virus envelope protein domain III, effectively reduces the risk of ADE in cases of infection with ZIKV and DENV (Sun et al., 2023). Increased focus on plant-based vaccines is warranted, as they offer a viable alternative version to the conventional VLP vaccine production systems. As an example, the plant-produced VLP COVID-19 vaccine Covifenz was authorized in Canada in 2022; however, the sponsor cancelled the authorization on March 31, 2023 (Su et al., 2023).

### 6.3 Additional innovations shaping next-generation vaccines

As depicted in a recent review paper by Eslami et al., 2025, next-generation viral vaccines are increasingly shaped by integrated technological pipelines that combine synthetic biology, nanotechnology, and advanced immunological design (Eslami et al., 2025). Innovative approaches often come from upgrading the existing development pathway through novel technologies such as codon optimization and engineered nucleic-acid constructs (mRNA/DNA), refined carrier systems, and precision antigen engineering guided by systems immunology and multi-omics profiling. For instance, while human codon optimization is widely used to enhance antigen expression, Cha et al. (2025) demonstrated that “viral-like” codon usage can further improve performance. By optimizing an mRNA–LNP vaccine encoding the neutralizing Gn-H domain of SFTSV using HSV-1 glycoprotein B (gB) codon usage, they achieved higher antigen expression, stronger humoral and cellular responses, increased bone marrow–resident antibody-secreting cells, and superior protection at lower mRNA doses compared with a human codon–optimized construct (Cha et al., 2025). This illustrates how innovation can arise not only from introducing new platform categories but also from refining core design variables.

Equally important are innovations that target enhancement of storage and intake, including thermostable formulations, needle-free delivery (e.g., microneedle patches and transcutaneous approaches), and mucosal or oral routes that may better align with transmission biology for certain respiratory and enteric viruses, while reducing cold-chain dependence and simplifying mass administration (Papadatou and Michos, 2025). Garcia-Valtanen et al. (2025) described a thermostable, unit solid-dose DNA vaccine format produced by lyophilization and compaction (sugar/sugar-alcohol/polymer matrix) that retained stability after 30 days across 4–42 °C and elicited robust antibody and T-cell responses with protection in a ZIKV NS1 model, highlighting how thermostable solid dosing can reduce cold-chain dependence and simplify administration for resource-limited settings (Garcia-Valtanen et al., 2025). Despite strong momentum, these advances still face practical barriers, which particularly include scalable manufacturing, standardized safety evaluation for novel delivery and adjuvant combinations, and equitable global access. Therefore, innovations that are not only scientifically sophisticated but also manufacturable, measurable, and deliverable at a population scale are needed in the future of vaccination (Eslami et al., 2025; Gupta et al., 2024).

## 7 Challenges and prospects of vaccination against viral infections

As previously noted, a variety of traditional and novel approaches have been introduced against viral infections. Vaccines have significantly reduced mortality from viral infections, contributing to the eradication of smallpox, the control of measles, and the management of the recent COVID-19 pandemic (Beladiya et al., 2024; Griffin, 2018; Meyer et al., 2020). However, managing viruses with vaccines is not always as simple as eradicating smallpox, and several problems arise during vaccine development. Table 1 summarizes the major vaccine platforms discussed in this review, highlighting their predominant immune response profiles, key advantages and limitations, and existing viral vaccine examples. Moreover, numerous viruses possess characteristics that complicate vaccine development. Viral agents, particularly emerging and re-emerging viruses, experience numerous and rapid genetic mutations (Shyr et al., 2021). The impact of these genetic alterations was observed during the COVID-19 pandemic, as the efficacy of vaccinations in controlling infections from the later variants, such as omicron, diminished (Gram et al., 2022). This decline occurred because the vaccines were developed for earlier strains of SARS-CoV-2 encountered in previous waves of the pandemic. This phenomenon has also been observed in other viruses, including influenza. Annually, there is a pressing need to develop new vaccines targeting circulating influenza strains, as antigenic changes in hemagglutinin and neuraminidase complicate the efficacy of vaccines against newly emerged strains (Gouma et al., 2020). Additionally, the persistent concern regarding the potential for an influenza pandemic due to antigenic shifts remains significant (Cargnin Faccin and Perez, 2024). Antigenic variability due to high mutation rates can, therefore, harden designing long-lasting strategies against viral pathogens. One proposed strategy is to design vaccines that focus immune responses on conserved epitopes, as suggested for HCV to address extensive sequence diversity and immune evasion (Pierce et al., 2020). Similarly, for SARS-CoV-2, variant-adapted immunogen engineering has been explored. For example, CoroVaxG.3-D.FR vaccine, an adenoviral vector-based vaccine designed to express variants of concern (VOC)-specific, membrane-bound spike stabilized in alternative prefusion conformations, has been reported to confer protection against various SARS-CoV-2 variants (Vinzón et al., 2023).

**Table 1:** **Immune response profiles and key attributes of major viral vaccine platforms.** ADE, antibody-dependent enhancement; ANDV, Andes virus; BSL, biosafety level; CCHFV, Crimean–Congo hemorrhagic fever virus; CDV, canine distemper virus; CHIKV, chikungunya virus; DENV, dengue virus; HAV, hepatitis A virus; HBV, hepatitis B virus; HCV, hepatitis C virus; HEV, hepatitis E virus; HIV-1, human immunodeficiency virus 1; HPV, human papillomavirus; HSV, herpes simplex virus; IAV-S, influenza A virus of swine; ILTV, infectious laryngotracheitis virus; JEV, Japanese encephalitis virus; LASV, Lassa virus; MARV, Marburg virus; NA, nucleic acid; NDV, Newcastle disease virus; OAS, original antigenic sin; RSV, respiratory syncytial virus; saRNA, self-amplifying RNA; SARS-CoV-2, severe acute respiratory syndrome coronavirus 2; TBEV, tick-borne encephalitis virus; VITT, vaccine-induced immune thrombotic thrombocytopenia; VLP, virus-like particle; VZV, varicella-zoster virus; ZEBOV, Zaire ebolavirus; ZIKV, Zika virus.

Platforms	Predominant Immunity Induced (Humoral / Cellular)	Advantages Mentioned	Limitations Mentioned	Viral Target Examples Mentioned
**Innactivated Vaccines**	Humoral	Safe, no chance of reactivation, thermostable, more accessible for underdeveloped regions, easy transportation	Low immunogenicity, short-term protection, low cellular immunity, need for booster doses, high manufacturing expenses, lower chance of vaccination adherence, need for high BSL for virus culture and inactivation, risk of ADE and OAS, rare risk of incomplete inactivation, use of chemical compounds for inactivation	HAV, influenza, JEV, TBEV, rabies virus, poliovirus
**Attenuated Vaccine**	Both	Systematic immune stimulation (natural infection mimicking), easy development, less or no need for booster doses	Secondary mutations and reactivation, not safe for immune-compromised individuals, inconclusive information on mechanisms underlying attenuation in traditional methods, need for cold chain transportation, need for trained administration providers, less accessible for underdeveloped regions, use of chemical compounds for attenuation in traditional methods	MMR (measle, mumps, rubella), ZEBOV, influenza, rotavirus, smallpox, JEV
**Protein Subunit, Multi-epitope, and Recombinant Protein Vaccines**	Humoral (also cellular if combined with novel delivery approaches)	Safe (no NA), low risk of harmful responses, high antibody titers, no chance of reversion, safe development (low BSL), specificity, high immunogenicity and efficacy	Need for multiple booster doses, low immunogenicity (without adjuvants), need for purification, empirical and labor-intensive target antigen selection	HBV, HPV, VZV, influenza, RSV
**Virus Like Particle Vaccines**	Both	Systematic immune stimulation (natural infection mimicking), safe (no NA), no inadvertent infection risk	Challenging manufacturing, need for purification, high cost of production, need for cold chain transportation, less accessible for underdeveloped regions	HBV, HPV, influenza, CHIKV, ZIKV
**RNA based Vaccine**	Both	High immunogenicity, potency, efficacy, and safety (no risk of integration), safe development (low BSL), no involvement of chemical compounds in production, rapid clinical development and mass production, low manufacturing expenses/ circRNA: (exonuclease tolerance)/ saRNA: long-term protection (due to replication)	Immunological responses, edema, thrombosis, instability, cold chain transportation, less accessible for underdeveloped regions, need for delivery systems, lack of knowledge on long-term side effects (ongoing research due to novelty of this platform)	SARS-CoV-2, HIV-1, Nipah virus, rabies virus, RSV, DENV, ZIKV, (circRNA: SARS-CoV-2), (saRNA: ZIKV, influenza, HSV, HIV, Ebola
**DNA based Vaccine (Delivery route plays an important role in outcome)**	Both	Thermostable, rapid clinical development and mass production, safe, High immunogenicity	Susceptible to anti-DNA immune responses, lack of optimal delivery systems, theorical risk of integration	ANDV, ILTV, rabies virus, TBEV, IAV-S, NDV, HEV, CDV, SARS-CoV-2, CCHFV
**Viral Vector Immunity (Delivery route and dose plays an important role in outcome)**	Both	Strong cellular immunity, potential for single dose immunization, no need for adjuvant or delivery systems, long-term protection (due to replication)	Pre-existing vector immunity (solution: non-human vectors like Chimpanzee or plant viral vectors), rare but severe adverse events (VITT), dose-dependent efficacy	SARS-CoV-2, ZEBOV, MARV, LASV, CCHFV, HCV, HSV-1 and 2, DENV

A further challenge is the infection enhancement resulting from the previous vaccination. ADE, for example, primarily occurs when non-neutralizing or sub-neutralizing antibodies facilitate viral entry into Fc receptor- or complement receptor-bearing cells, enhancing infection and sometimes triggering excessive inflammatory responses. ADE has been implicated in the failure or restricted use of some vaccines, such as Dengvaxia for dengue, where vaccination of seronegative individuals increased the risk of severe disease upon subsequent infection. There is an increasing concern regarding the COVID-19 vaccines and the potential for similar issues. A study showed a lower risk of ADE after human vaccination with mRNA SARS-CoV-2 vaccines compared to inactivated or adenovirus-vector vaccines (Luo et al., 2025; Tan et al., 2025; Thomas et al., 2025). However, no conclusive evidence is still in hand. Moreover, an RSV vaccination was previously linked to an increase in lung infection known as vaccine-associated enhanced respiratory disease (VAERD) (Diamond and Pierson, 2020; Ikewaki et al., 2023). Given these risks, multiple antigen design strategies have been proposed to minimize ADE and related immune enhancement. These include focusing immune responses on neutralizing, type-specific epitopes (e.g., DENV envelope domain III), engineering immunogens to avoid exposure of ADE-prone regions (e.g., fusion loop epitopes), and modifying antibody Fc regions to reduce Fc receptor binding. Structure-guided and epitope-focused vaccine approaches are being developed for DENV, ZIKV, SARS-CoV-2, and other viruses (Shukla et al., 2020; Slon-Campos et al., 2019a; Wei et al., 2025). A study on the ADE challenge demonstrated that a ZIKV E-dimer-based subunit vaccine, which eliminates the fusion loop epitope of the prM and E proteins, can generate antibodies highly specific to the E-dimer of ZIKV. The antibodies generated through ZIKV E-dimer immunization exhibit no cross-reactivity with DENV, representing a crucial advancement in preventing ZIKV-related ADE, as no poorly neutralizing antibodies associated with ADE are produced (Slon-Campos et al., 2019b). In addition, non-neutralizing S2-specific antibodies elicited by alphavirus-vectored vaccines confer protection against lethal heterologous disease outcomes in models challenged with SARS-CoV-2 and clade 2 bat sarbecovirus (Adams et al., 2023).

**Fig. 1:**
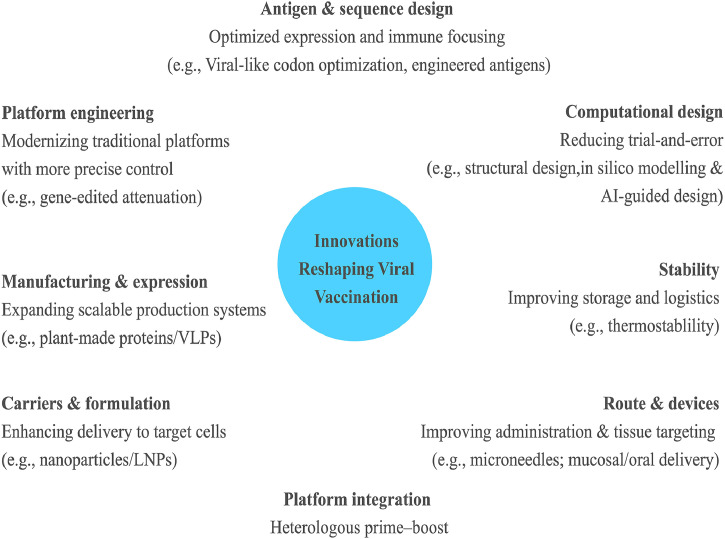
Innovations reshaping viral vaccination. AI, artificial intelligence; LNP, lipid nanoparticle; VLP, virus-like particle.

As discussed in the previous sections, the future of vaccination against viral infections is increasingly linked to precision and specificity, supported by technologies that can reduce reliance on lengthy and costly experimental iteration. In silico modelling, structural vaccinology, and computational immunogen design can help prioritize candidates, anticipate antigenic drift, and refine vaccine constructs before extensive wet-lab and in vivo testing. These approaches are particularly valuable for viruses with high mutation rates and pronounced antigenic variability, and they are expected to play a central role in the rational development of broader and potentially universal vaccines. Importantly, this shift does not imply abandoning traditional vaccine approaches; rather, it enables established platforms to be strengthened through more rational antigen selection and optimization, improved delivery and formulation strategies, and more efficient manufacturing and evaluation pipelines. Combining platforms can also help offset the limitations of any single approach. For example, heterologous prime–boost regimens (e.g., an inactivated vaccine prime followed by an mRNA booster) can enhance both humoral and cellular immune responses by leveraging complementary immunological strengths across platforms (Zuo et al., 2022). Together, integrating these computational tools with proven platform experience may accelerate the development of vaccines that are not only effective but also safer, more scalable, and more deployable in diverse real-world settings. Fig. 1 provides an overview of the principal innovations reshaping viral vaccination, as discussed in this review.

## Funding

This work was supported by 10.13039/501100003093Ministry of Higher Education, Malaysia for niche area research under the Higher Institution Centre of Excellence (HICoE) program (MO002-2019 and TIDREC-2023).

## Data availability statement

Data sharing not applicable to this article as no datasets were generated or analysed during the current study.

## CRediT authorship contribution statement

**Niloofar Farsiu:** Visualization, Writing – original draft, Writing – review & editing. **Fatemeh Khodadadpour Mahani:** Writing – original draft. **Nasir Arefinia:** Writing – original draft. **Javad Charostad:** Writing – original draft, Writing – review & editing. **Mohammad Pardeshenas:** Writing – original draft. **Habibollah Mirzaei:** Writing – review & editing. **Mohsen Nakhaie:** Conceptualization, Supervision, Writing – review & editing. **Pouya Hassandarvish:** Supervision, Writing – review & editing. **Sazaly AbuBakar:** Supervision, Writing – review & editing.

## Declaration of competing interest

The authors declare that they have no known competing financial interests or personal relationships that could have appeared to influence the work reported in this paper.
